# Life and disease status of patients with Parkinson’s disease during and after zero-COVID in China: an online survey

**DOI:** 10.1186/s40035-024-00399-9

**Published:** 2024-02-06

**Authors:** Pei Huang, Yu-Yan Tan, Biao Chen, Hui-Fang Shang, Li-Juan Wang, Chun-Feng Liu, Ling Chen, Ying Chang, Han Wang, Xue-Lian Wang, Xiao-Guang Lei, Li-Fen Yao, Yang Yu, Zheng Ye, Hai-Bo Chen, Sheng-Di Chen

**Affiliations:** 1 Department of Neurology and Institute of Neurology, Ruijin Hospital, Shanghai Jiao Tong University School of Medicine, Shanghai, 200025 China; 2 Department of Neurology, Xuan Wu Hospital Affiliated to Capital Medical University, Beijing, 100053 China; 3 Department of Neurology, West China Hospital, Sichuan University, Chengdu, 610041 China; 4 Department of Neurology, Guangdong General Hospital, Guangzhou, 510080 China; 5 Department of Neurology, Suzhou Clinical Research Center of Neurological Disease, The Second Affiliated Hospital of Soochow University, Suzhou, 215004 China; 6 Department of Neurology, The First Affiliated Hospital of Sun Yat-Sen University, Guangzhou, 510080 China; 7 Department of Neurology, China-Japan Union Hospital, Jilin University, Changchun, 130031 China; 8 Department of Neurology, Peking Union Medical College Hospital, Chinese Academy of Medical Sciences and Peking Union Medical College, Beijing, 100730 China; 9 Department of Neurosurgery, West China Hospital, Sichuan University, Chengdu, 610041 China; 10 Department of Neurology, The First Affiliated Hospital of Kunming Medical University, Kunming, 650032 China; 11 Department of Neurology, The First Affiliated Hospital of Harbin Medical University, Harbin, 150001 China; 12 Department of Neurology, Tianjin Huanhu Hospital, Tianjin, 300350 China; 13 Institute of Neuroscience, Center for Excellence in Brain Science and Intelligence Technology, Chinese Academy of Sciences, Shanghai, 200031 China; 14 Department of Neurology, Beijing Hospital, National Center of Gerontology, Beijing, 100730 China; 15 Lab for Translational Research of Neurodegenerative Diseases, Shanghai Institute for Advanced Immunochemical Studies (SIAIS), Shanghai Tech University, Shanghai, 201210 China

**Keywords:** COVID-19, Parkinson’s disease, Lockdown, Infection, Zero-COVID

## Abstract

**Background:**

Little is known about the impact of the COVID-19 pandemic on patients with Parkinson’s disease (PD) at different stages of the pandemic. This study aims to assess the lives and disease status of PD patients during the zero-COVID policy period and after ending the zero-COVID policy.

**Methods:**

This multicenter cross-sectional study included two online surveys among PD patients in China, from May 30 to June 30 in 2022 and from January 1 to February 28 in 2023, respectively. The survey questionnaires contained four sections: (1) status of COVID-19 infection; (2) impact on motor and non-motor symptoms; (3) impact on daily and social lives; and (4) impact on PD disease management.

**Results:**

A total of 1764 PD patients participated in the first online survey, with 200 patients having lockdown experience and 3 being COVID-19-positive (0.17%). In addition, 537 patients participated in the second online survey, with 467 patients having COVID-19 infection (86.96%). (1) During zero-COVID, all of the COVID-19-positive patients had mild symptoms of COVID-19 and no death was reported. After zero-COVID, 83.51% of the COVID-19-positive patients had mild symptoms. The overall death rate and inpatient mortality rate of COVID-19-positive PD patients were 3.21% and 30.00%, respectively. (2) During zero-COVID, 49.43% of PD patients reported worsening of PD-related symptoms (lockdown vs. unlockdown, 60.50% vs. 48.02%, *P* = 0.0009). After zero-COVID, 54.93% of PD patients reported worsening of PD-related symptoms (COVID-19 positive vs. COVID-19 negative, 59.31% vs. 25.71%, *P* < 0.0001). (3) During zero-COVID, 62.36% of patients felt worried, and ‘limited outdoor activities’ (55.39%) was the top reason for mental health problems. After zero-COVID, 59.03% of patients felt worried, with ‘poor health’ (58.10%) being the top reason. The PD patients tended to change their daily activities from offline to online, and their economic and caregiver burdens increased both during and after zero-COVID. (4) Most PD patients would like to choose online rehabilitation during (69.56%) and after zero-COVID (69.27%). The demand for online medication purchasing also increased during (47.00%) and after zero-COVID (26.63%).

**Conclusions:**

The COVID-19 pandemic aggravated the motor and non-motor symptoms of PD patients either during or after the zero-COVID policy period. The PD patients also experienced prominent mental health problems, changes in daily activities, and increases in economic and caregiver burdens. The COVID-19 pandemic has changed ways of PD management with increasing demands for online medication purchasing and rehabilitation.

**Supplementary Information:**

The online version contains supplementary material available at 10.1186/s40035-024-00399-9.

## Introduction

The coronavirus disease 2019 (COVID-19) pandemic caused by the severe acute respiratory syndrome coronavirus 2 (SARS-CoV-2) has quickly spread globally, causing threats and troubles to the lives of humans all over the world. To limit the spreading of the virus, many countries introduced a series of measures including mask wearing, limits on public and private gatherings, restrictions on domestic movement and public transport, stay-at-home orders, etc. [1]. In China, the dynamic zero-COVID policy was adopted based on the level of risks in different areas, and different levels of restriction were imposed accordingly [2]. During the zero-COVID period, the lockdown measures might have effectively prevented the spread of the virus and protected people from infection. However, it also led to the stagnation of social and economic activities. Persons with chronic diseases, including Parkinson’s disease (PD), might have experienced lifestyle changes, psychological stress, and symptom aggravation.

With the gradual formation of the COVID-19 immune barrier, the increased awareness of virus prevention, and the advancement in COVID-19 treatment, a sequential re-opening policy was adopted in China on December 7th, 2022 [3]. After that, peaks of COVID-19 infection gradually emerged in China. As PD patients are reported to be vulnerable to COVID-19 infection [4], they might experience symptom worsening and have increased risks of hospitalization and death with the emerging peak of COVID-19 epidemic. An online survey in USA found that PD patients without COVID-19 had worsened motor (43%) and non-motor (52%) symptoms during the pandemic [5]. Among PD patients with COVID-19, 55% reported worsening of at least one existing motor symptom [5]. Worsening of non-motor symptoms was also noted, including mood symptoms (71%), sleep disruptions (62%), cognitive problems (49%), and dysautonomia (38%) [5]. A cross-sectional study of 5,210,432 inpatients from 1468 hospitals in Germany has collected data from 64,434 PD patients with 693 being COVID-19-positive [6], and found that the COVID-19 inpatient mortality rate was much higher in PD patients than in non-PD patients (35.4% vs. 20.7%) [6]. A cohort study by Vignatelli and colleagues assessed the risk of hospitalization for and death from COVID-19 in 696 PD patients compared with 8590 controls. The 3-month hospitalization rate for COVID-19 was 0.6% in PD and 0.7% in controls. The 30-day risk of death after hospitalization was high in both PD patient group and control group (around 35%) without a difference between the two groups [7]. COVID-19 might affect the status of PD patients [8]. To our interest, there were two phases of the pandemic in China, i.e., during the zero-COVID policy period and after ending the zero-COVID policy. Whether the change of policy would lead to different characteristics of the impact of COVID-19 on PD patients is worthy of exploring.

In addition, the COVID-19 pandemic had a major impact on healthcare utilization, such as reductions in hospital visits and some selective increases in online health services due to the measures such as lockdown and stay-at-home orders [9]. The application of telemedicine, such as electronic prescribing, medical consults, and online rehabilitation, maintained the delivery of routine health care to patients with chronic neurological diseases [10]. However, there was no comprehensive investigation into how the way PD patients receive medical care changed, and what impacted the availability of telemedicine in PD patients during the COVID-19 epidemic in China. Understanding the change of the way by which PD patients received medical care during the pandemic, may provide insights into the planning of healthcare resources during the outbreak of infectious diseases in the future.

In this study, we set out to understand how the COVID-19 pandemic affected PD patients during and after zero-COVID in China. We evaluated the effects of COVID-19 pandemic on the motor and non-motor symptoms, daily and social lives, healthcare management, as well as COVID-19 infection-related symptoms, hospitalization, and death in PD patients.

## Methods

### Study design

This multicenter study included two online surveys of PD patients in China, conducted during and after the zero-COVID period, respectively. Questionnaires were collected from May 30 to June 30, 2022 to assess the status of PD patients during zero-COVID, with lockdown measures as a national policy to prevent COVID-19 spread in China. ‘Lockdown’ was defined as an experience of lockdown management with a duration longer than or equal to 2 weeks. To evaluate the status of PD patients after zero-COVID, a second questionnaire survey was conducted from January 1 to February 28, 2023. Ethical approval was obtained from the ethics committee of Ruijin Hospital Affiliated with Shanghai Jiao Tong University School of Medicine. Participants provided consent to participate via an online consent form before the start of the survey.

### Questionnaire design

The survey questionnaires mainly contained four sections: (1) status of COVID-19 infection among PD patients; (2) impact of COVID-19 on PD motor and non-motor symptoms; (3) impact of COVID-19 on PD daily and social lives; and (4) impact of COVID-19 on PD disease management (first survey: https://jinshuju.net/f/LpNDjz, second survey: https://jinshuju.net/f/uIDP0D). For details, please see Additional file 1: Method S1.

### Statistical analysis

Survey results and demographic information were summarized using descriptive statistics. Differences in means of continuous variables were analyzed with Student’s *t*-test for normally distributed data and Wilcoxon rank sum for non-normally distributed. Comparison of categorical variables was performed with the chi-square test. To analyze quantitative and qualitative variables, *t*-test for independent samples (two-tailed) and chi-square test were performed, respectively. Significance was fixed at 0.05. All analyses were carried out with the SPSS v.26 software.

## Results

### The demographics of PD participants

**Fig. 1:**
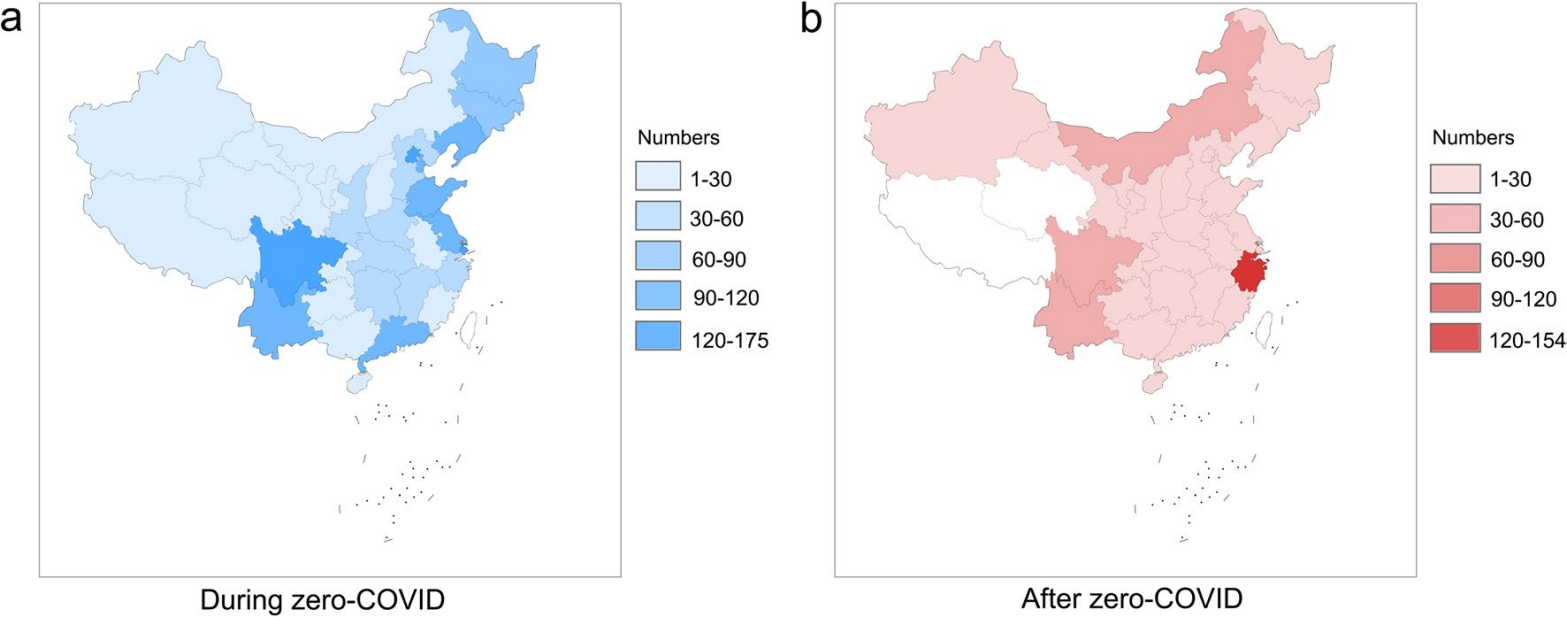
Distribution of participants in provincial administrative regions in China in the survey during zero-COVID (**a**) or after zero-COVID (**b**)

A total of 1764 PD patients from 32 provinces in China (Fig. 1a, Additional file 1: Table S1) participated in the online survey during zero-COVID, of whom 200 PD patients reported lockdown experience. Only 3 PD patients were diagnosed as COVID-19-positive (0.17%). In addition, 537 PD patients from 29 provinces (Fig. 1b, Additional file 1: Table S2) participated in the online survey after zero-COVID, with 467 patients having COVID-19 infection (86.96%).

### General status of COVID-19 infection among PD patients

**Fig. 2:**
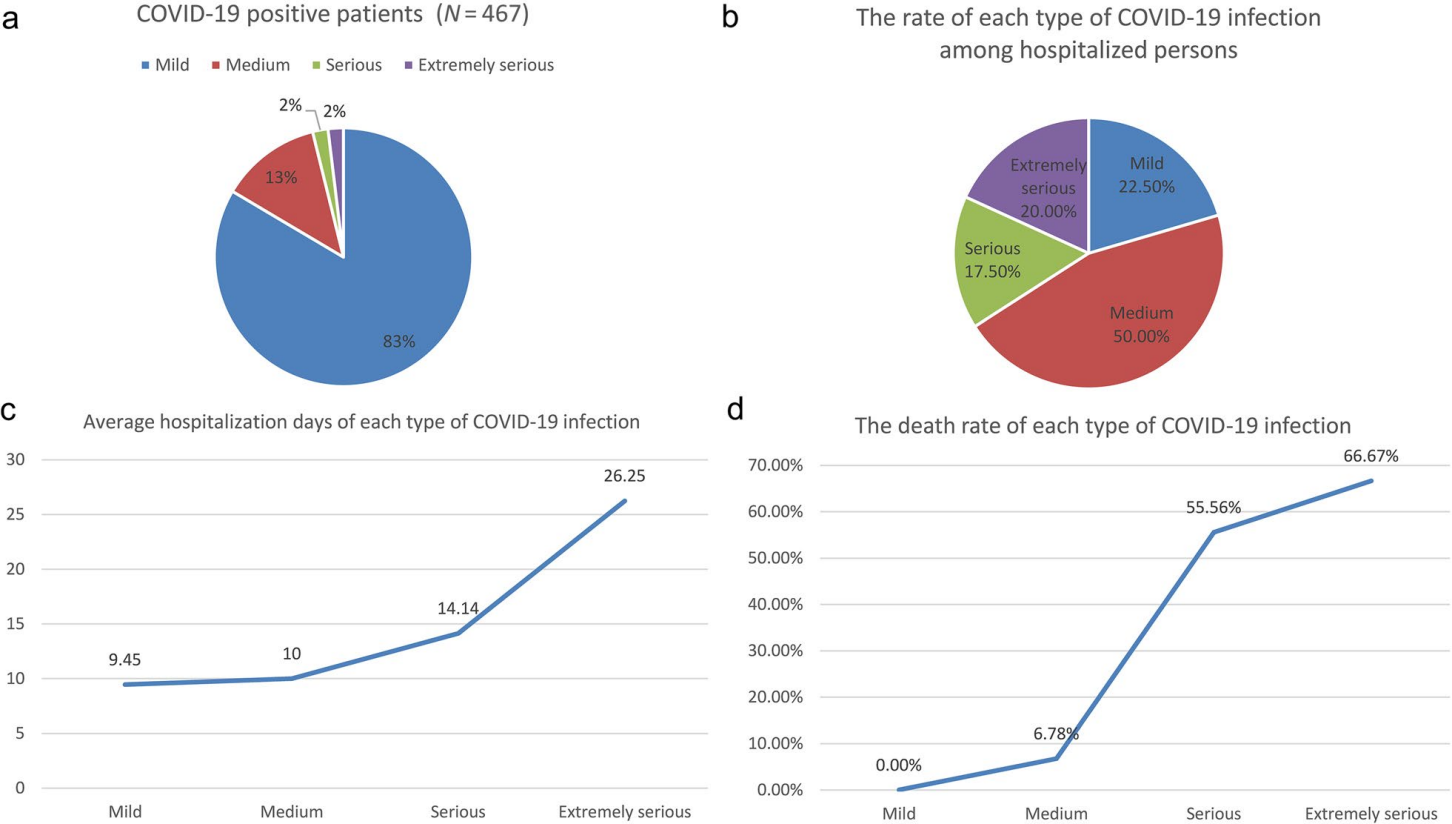
Hospitalization days and death rate of COVID-19-positive PD patients after zero-COVID. **a** The proportions of patients with different severities of COVID-19 among the whole cohort. **b** The proportions of patients with different severities of COVID-19 among hospitalized patients. **c** Average length of hospitalization of PD patients with different severities of COVID-19. **d** The death rate of PD patients with different severities of COVID-19

During zero-COVID, only 3 PD patients were diagnosed as COVID-19-positive (0.17%). The three patients were transferred to Central Hospital for Public Health immediately after diagnosis. They were classified as mild type of COVID-19 infection and no death was reported. After zero-COVID, 86.96% of PD participants were COVID-19-positive, among whom 83.51% were mild, 12.63% were medium, 1.93% were serious and 1.93% were extremely serious (Fig. 2a). The most commonly reported symptoms after COVID-19 infection were cough (73.66%), fever (66.60%), weakness (61.03%), fatigue (56.96%) and sore throat (46.90%), which were respiratory symptoms. Systemic symptoms and problems of digestive and circulatory systems were also reported. Other symptoms included dizziness, insomnia, sweating, delirium, etc. (Additional file 1: Fig. S1). In addition, 40 out of the 467 COVID-19-positive PD patients (8.57%) had the experience of hospitalization. Among the inpatient PD patients, 22.50% were mild, 50.00% were medium, 17.50% were serious and 20.00% were extremely serious (Fig. 2b). The average length of hospital stay significantly increased with the severity of the disease (from mild 9.45 days to extremely serious 26.25 days) (Fig. 2c). Fifteen COVID-19-positive PD patients (3.21%, 12 inpatients and 3 outpatients) died after COVID-19 infection, and the inpatient mortality rate of COVID-19-positive PD patients was 30.00%. The death rate also increased significantly with the severity of the disease (from medium 6.78% to extremely serious 66.07%) (Fig. 2d).

### Impact of the COVID-19 pandemic on PD motor and non-motor symptoms

During zero-COVID, 49.43% of 1764 PD patients reported worsening of PD symptoms (60.50% with lockdown experience vs. 48.02% without lockdown experience, *χ*^2^ = 11.05, *P* = 0.0009). Age stratification analysis showed that the proportion of patients with worsening of symptoms was highest in those aged ≤ 50 years (57.14% in ≤ 50 years, 47.20% in 51–65 years, 50.43% in 66–75 years, and 45.63% in > 75 years). After zero-COVID, 54.93% of the 537 PD patients reported worsening of symptoms (59.31% for COVID-19 positive vs. 25.71% for COVID-19 negative, *χ*^2^ = 27.76, *P* < 0.0001). The proportion of patients with worsening of symptoms was highest in those aged > 75 years (52.38% in ≤ 50 years, 51.34% in 51–65 years, 56.93% in 66–75 years, and 66.67% in > 75 years).

**Fig. 3:**
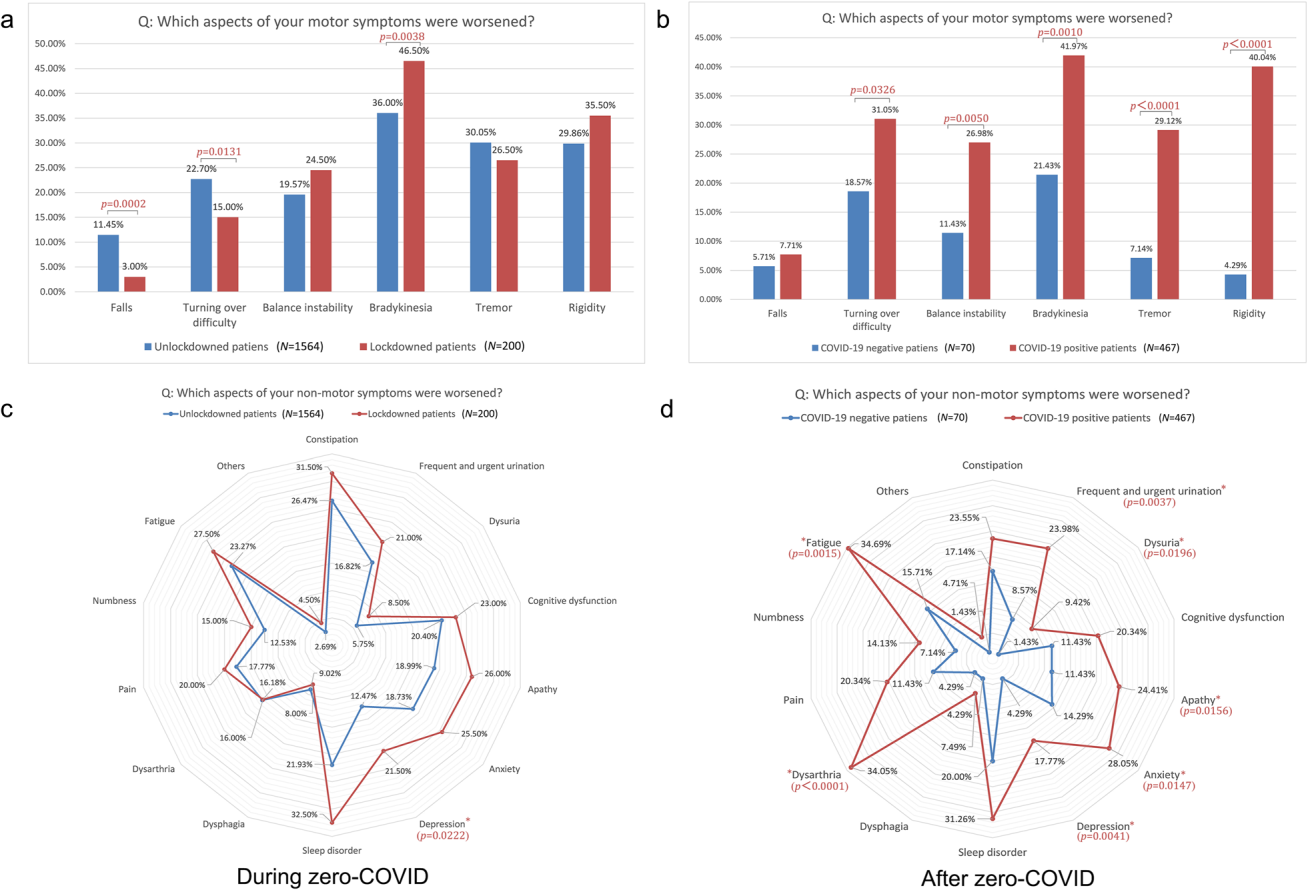
The worsened motor and non-motor symptoms in PD patients during and after zero-COVID. **a** The worsened motor symptoms in PD patients with or without lockdown experience during zero-COVID. **b** The worsened motor symptoms in COVID-19-positive and negative PD patients after zero-COVID. **c** The worsened non-motor symptoms in PD patients with or without lockdown experience during zero-COVID. **d** The worsened non-motor symptoms in COVID-19-positive and negative PD patients after zero-COVID. Significant differences between lockdowned and unlockdowned PD patients, and between COVID-19-positive and negative PD patients in motor and non-motor symptoms are labeled with *P* values

During zero-COVID, the top three aggravated motor symptoms among the patients with lockdown experience were bradykinesia (46.50%), rigidity (35.50%) and tremor (26.50%), while bradykinesia (36.00%), tremor (30.05%) and rigidity (29.86%) were prominent among the PD patients without lockdown experience (Fig. 3a). After zero-COVID, the top three aggravated motor symptoms among the COVID-19-positive patients were bradykinesia (41.97%), rigidity (40.04%) and turning over difficulty (31.05%), while bradykinesia (21.34%), turning over difficulty (18.57%) and balance instability (11.43%) were obvious among the COVID-19-negative PD patients (Fig. 3b).

As for non-motor symptoms, during zero-COVID, the top five worsened non-motor symptoms among the patients with lockdown experience were sleep disorder (32.50%), constipation (31.50%), fatigue (27.50%), apathy (26.00%) and anxiety (25.50%), while those among the PD patients without lockdown experience were constipation (26.47%), fatigue (23.27%), sleep disorder (21.93%), cognitive dysfunction (20.40%) and apathy (18.99%) (Fig. 3c). After zero-COVID, the top five worsened non-motor symptoms were fatigue (34.69%), dysarthria (34.05%), sleep disorder (31.26%), anxiety (28.05%) and apathy (24.41%) among COVID-19-positive patients, while those among the COVID-19 negative PD patients were sleep disorder (20.00%), constipation (17.24%), fatigue (15.71%), anxiety (14.29%) and apathy (11.43%) (Fig. 3d).

### Impact of the COVID-19 pandemic on daily and social lives of PD patients

#### Mental psychology

**Fig. 4:**
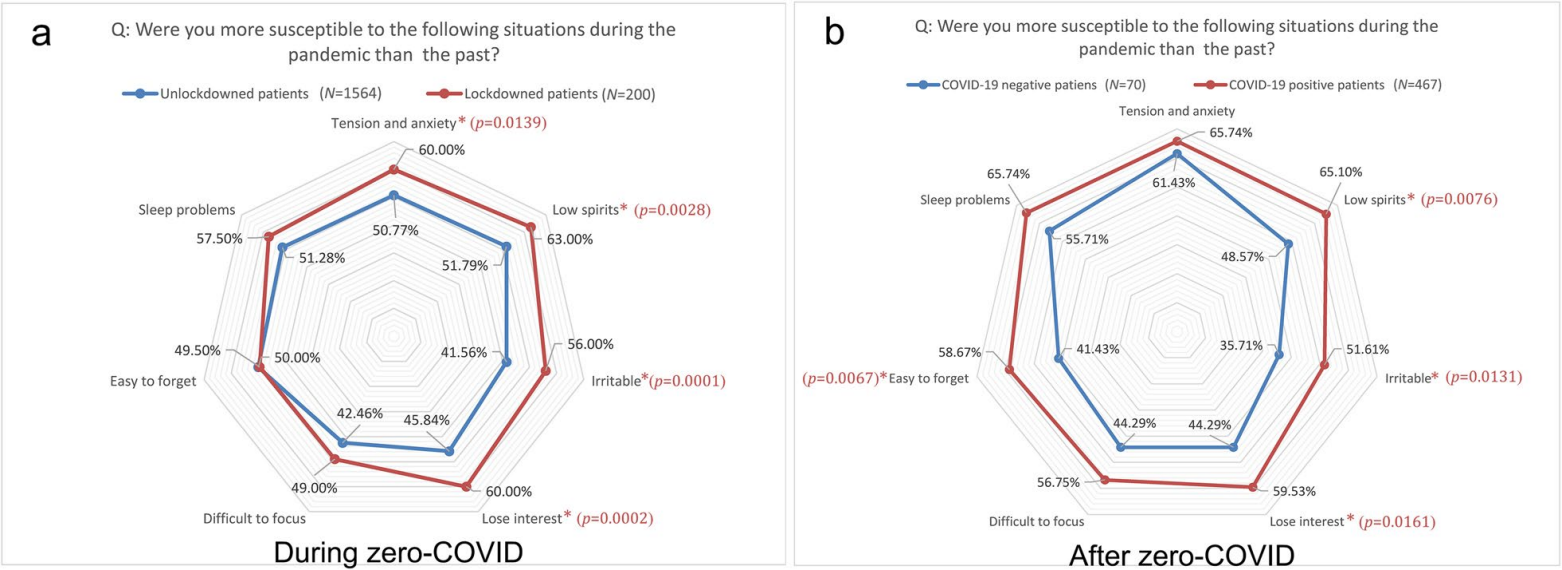
The mental psychological problems among PD patients during and after zero-COVID. **a** The mental psychological problems in PD patients with or without lockdown experience during zero-COVID. **b** The mental psychological problems in COVID-19-positive and negative PD patients after zero-COVID. Significant differences in mental psychological problems between PD patients with and without lockdown experience, and between COVID-19-positive and negative PD patients  are labeled with *P* values

The COVID-19 pandemic not only affected the symptoms, but also impacted the daily and social lives of PD patients. The proportions of patients who felt worried about the pandemic were 62.36% during zero-COVID and 59.03% after zero-COVID (Additional file 1: Fig. S2). During zero-COVID, PD patients were likely to have mental health problems such as low spirits (53.06%), sleep problems (51.98%), tension and anxiety (51.81%). The patients with lockdown experience seemed to be more susceptible to low spirits (63.00% vs. 51.79%, *χ*^2^ = 8.946, *P* = 0.0028), tension and anxiety (60.00% vs. 50.77%, *χ*^2^ = 6.054, *P* = 0.0139), losing interest (60.00% vs. 45.84%, *χ*^2^ = 14.25, *P* = 0.0002), and irritability (56.00% vs. 41.56%, *χ*^2^ = 15.07, *P* = 0.0001) than the patients without lockdown experience (Fig. 4a).

**Fig. 5:**
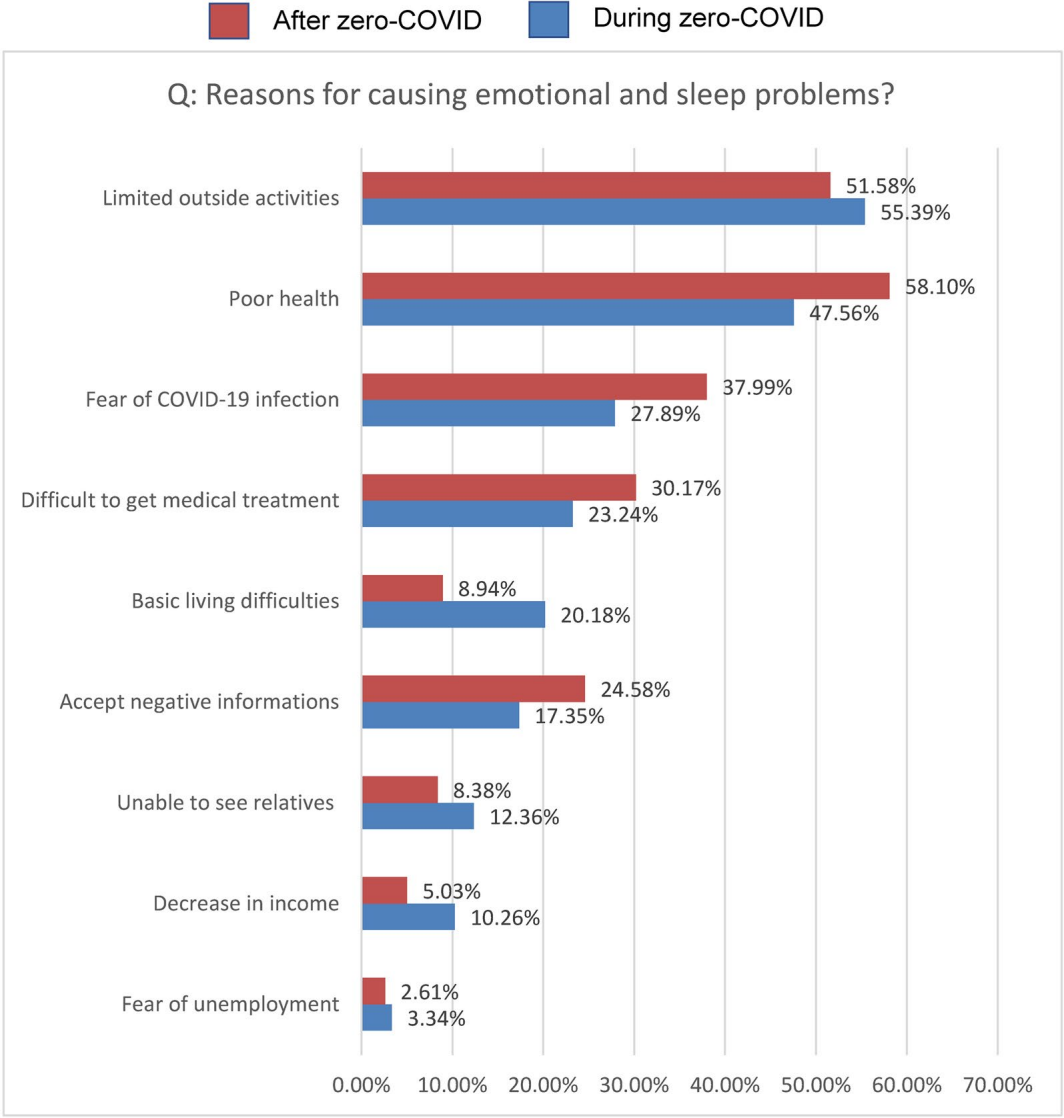
Reasons for emotional and sleep problems among PD patients during and after zero-COVID

After zero-COVID, the PD patients were likely to have mental health problems such as tension and anxiety (65.18%), sleep problems (64.43%), and low spirits (62.94%). The COVID-19-positive patients seemed to be more susceptible to low spirits (65.10% vs. 48.57%, *χ*^2^ = 7.127, *P* = 0.0076), irritability (51.61% vs. 35.71%, *χ*^2^ = 6.150,* P* = 0.0131), losing interest (59.53% vs. 44.29%, *χ*^2^ = 5.790, *P* = 0.0161), and being easy to forget (58.67% vs. 41.43%, *χ*^2^ = 7.362, *P* = 0.0067) than the COVID-19-negative patients (Fig. 4b). Limited outdoor activities (55.39%) and poor health (58.10%) were the top reason for mental health problems during and after zero-COVID, respectively (Fig. 5).

#### Daily activity

The PD patients reported reduced physical exercise during the pandemic (37.50% during zero-COVID vs. 62.76% after zero-COVID,* χ*^2^ = 109.00, *P* < 0.0001) with increases in online contacts and activities to compensate for the decreased offline activities. A large proportion of PD patients reported reduced short-distance trips, long-distance trips, community activities and indoor activities, both during zero-COVID and after zero-COVID. However, a proportion of PD patients had increased online disease education (23.92% during zero-COVID, 17.13% after zero-COVID) and online patient communication (22.34% during zero-COVID, 15.27% after zero-COVID) during the pandemic (Additional file 1: Figs. S3 and S4).

#### Economic and caregiver burdens

A large proportion of PD patients had decreased total household income (38.53% during zero-COVID, 31.47% after zero-COVID) and patient’s own income (26.21% during zero-COVID, 19.74% after zero-COVID). However, high proportions of PD patients had remarkably increased total household expenditure (44.40% during zero-COVID, 49.53% after zero-COVID) and their medical expenditure (40.41% during zero-COVID, 48.42% after zero-COVID). High proportions of PD patients (34.92% during zero-COVID, 45.62% after zero-COVID) complained about the increased demands for care from caregivers (Additional file 1: Fig. S5).

### Impact of the COVID-19 pandemic on PD disease management

#### Allopatric medical treatment

The COVID-19 pandemic had changed the form of medical treatment and the strategy of disease management for PD patients. Before the COVID-19 pandemic, 38.49% of PD patients received allopatric medical treatment (Additional file 1: Fig. S6a), among whom only 11.49% kept allopatric medical care as usual whereas 68.78% reported reduced frequency of allopatric medical treatment during zero-COVID (Additional file 1: Fig. S6b). After zero-COVID, only 12.29% of PD patients kept allopatric medical care as usual whereas 24.02% of patients reduced their frequency of allopatric medical treatment. Most (63.69%) of the PD patients kept local medical care as usual after zero-COVID (Additional file 1: Fig. S6b).

#### Online and offline medical treatment

A large number of PD patients stopped offline outpatient clinic visiting (24.60% during zero-COVID, 26.82% after zero-COVID) or reduced the frequency of offline outpatient clinic visiting (34.52% during zero-COVID, 20.11% after zero-COVID) during the pandemic. Many patients switched to online medical consultation (56.24% during zero-COVID, 49.16% after zero-COVID) with most patients (88.28% during zero-COVID, 87.12% after zero-COVID) considering that their problems could be solved online (Additional file 1: Fig. S7, Fig. S8, Fig. S9).

#### Online rehabilitation

Many patients (69.56% during zero-COVID, 69.27% after zero-COVID) would like to choose remote rehabilitation guidance online. Among the patients having online rehabilitation experience (8.22% during zero-COVID vs. 18.81% after zero-COVID, *χ*^2^ = 237.0, *P* < 0.0001), most found it helpful (81.38% during zero-COVID, 52.48% after zero-COVID) and were willing to continue online rehabilitation (74.48% during zero-COVID, 76.53% after zero-COVID) (Additional file 1: Fig. S10, Fig. S11, Fig. S12).

#### Online medication purchasing

During zero-COVID, 47.00% of patients exhibited increased demands for online medication purchasing, and 12.00% were completely dependent on it. The patients with lockdown experience showed a higher demand than the patients without lockdown experience (increased demand: 49.50% vs. 46.68%, completely dependent: 16.50% vs. 11.45%, *χ*^2^ = 6.76, *P* = 0.034) (Additional file 1: Fig. S13a). After zero-COVID, only 26.63% of patients exhibited increased demands for online medication purchasing, and 8.01% of patients were completely dependent on it, while 65.36% had no demand for online medication purchasing (Additional file 1: Fig. S13b).

## Discussion

### Vulnerability and prognosis of COVID-19 infection in PD patients

Our study found that the infection rate of COVID-19 during and after zero-COVID were 0.17% and 86.96%, respectively. During zero-COVID, all the COVID-19-positive PD patients were mild and no death was reported. After zero-COVID, 83.51% of COVID-19-positive PD patients were mild. The overall hospitalization and death rates among COVID-19-positive patients were 8.57% and 3.21%, respectively. The inpatient mortality rate of COVID-19-positive PD patients was 30.00%. The average length of hospitalization and the death rate gradually increased with aggravation of COVID-19 severity.

As for the overall death rate, a multicenter study of 117 community-dwelling PD patients with COVID-19 in Italy, Iran, Spain, and the UK reported an overall mortality of 19.7% [11]. Our study reported a lower overall death rate of 3.21% in the COVID-19-positive patients. The difference may be partly due to the older age in their study compared to ours (71.4 ± 10.8 years vs. 63.45 ± 10.04 years). In fact, older age is consistently found to be a major risk factor for death in patients with COVID-19 [12]. As for the inpatient mortality rate, a retrospective review of 70 PD inpatients in New York found that PD patients with COVID-19 infection (mean age 78.7 years, 58.5% male) had a higher mortality rate compared to those not infected (35.8% vs. 5.9%) [13]. A study with a larger cohort of 552 COVID-19-positive PD inpatients (mean age 80 years, 66% male) reported a 34-day mortality rate of 38.4% [14]. Another study with a large cothort of 64,434 PD patients with 693 being COVID-19-positive (mean age 80.8 years, 60.5% male) showed an inpatient mortality rate of 35.4% [6]. Our study showed an inpatient mortality rate of 30.00%, which was lower than or similar to the previously reported data. This may be due to the much younger age (63.45 ± 10.04 years) of PD patients and smaller proportion of male patients (51.82% male) in our study, as old age and male sex are both risk factors for death among COVID-19 patients [12, 15]. Moreover, some other confounding factors such as comorbidities and medical levels may also influence the mortality rate of COVID-19 patients [12]. In addition, the online survey may lead to a bias towards milder COVID-19 cases as the patients who were able to complete a questionnaire may have recovered from the infection. There was a possibility of underestimating the proportion of death and severe disease in this study. The zero-COVID policy might have delayed the death rather than reducing it. It might have given us enough time to be prepared for the epidemic and to avoid the healthcare system becoming overwhelmed at the first shock all over the country. However, we have to interpret our research results cautiously due to the limitations of our study.

### Effect of COVID-19 on motor and non-motor symptoms in PD patients

Many PD patients reported worsening of symptoms (49.43% during zero-COVID vs. 54.93% after zero-COVID) during the pandemic. During zero-COVID, patients aged ≤ 50 years had a higher proportion of worsened symptoms (57.14%). PD patiens with lockdown experience (60.50%) had higher rate of worsening than those without (48.02%). After zero-COVID, patients aged > 75 years had a higher rate of worsened symptoms (66.67%). The infected PD patients (59.31%) had a higher proportion of deterioration than uninfected patients (25.71%). We inferred that the symptoms of PD could be worsened directly and indirectly by the COVID-19 pandemic. During zero-COVID, lockdown might have a greater impact on younger patients due to a marked reduction in physical activities. After zero-COVID, infection might be more harmful to older patients due to their poor health status.

During zero-COVID, bradykinesia was the most common aggravated motor symptom among both patients with and without lockdown experience (46.50% and 41.97%, respectively), and the most obvious aggravated non-motor symptoms among them were sleep disorder (32.50%) and constipation (26.47%), respectively. After zero-COVID, the most common aggravated motor symptoms among positive and negative patients were also bradykinesia (41.97% and 21.34%, respectively), and the most obvious aggravated non-motor symptoms among them were fatigue (34.69%) and sleep disorder (20.00%), respectively.

Infection is a common cause of exacerbation of parkinsonian symptoms [16]. A severe infection such as COVID-19 could have a direct harmful impact on PD motor symptoms, which deteriorate during systemic inflammation [17]. A large online study (Fox Insight) has investigated the effects of the COVID-19 pandemic on PD patients [5]. Data were collected from 5429 PD patients with 51 reporting COVID-19 positive diagnoses in the US. Among those infected, 55% reported worsening of at least one existing motor symptom [5]. Worsening of non-motor symptoms was also noted, including mood symptoms (71%), sleep disruptions (62%), cognitive problems (49%), and dysautonomia (38%) [5]. However, the underlying mechanisms of the direct harmful effect of COVID-19 on PD are still unclear. Possible explanations encompass insufficient response to dopaminergic drugs after infection, altered transport of dopaminergic drugs through the blood–brain barrier after systemic infections, and altered dopamine metabolism and receptor signaling due to the response to proinflammatory cytokines. Inflammatory processes located outside the brain may boost the pre-existing neuroinflammatory processes in PD [18, 19].

COVID-19 also affects motor and non-motor symptoms of PD through indirect ways, such as dramatic changes in routine life, social isolation, stress, anxiety and prolonged immobility [20]. In the Fox Insight online study, PD patients without COVID-19 reported worsened motor (43%) and non-motor (52%) symptoms during the pandemic [5]. This phenomenon might be explained by a marked reduction in physical activities, increased psychological stress, and difficulties in getting access to dopaminergic drugs or medical care due to shutdowns [20].

### Effect of COVID-19 on daily and social lives in PD patients

During zero-COVID, 62.36% of patients felt worried. Patients with lockdown experience seemed to be more susceptible to low spirits (63.00% vs. 51.79%), tension and anxiety (60.00% vs. 50.77%), and loss of interest (60.00% vs. 45.84%) than the patients without lockdown experience. ‘Limited outdoor activities’ (55.39%) was the top reason for mental health problems. After zero-COVID, 59.03% of the patients felt worried. The COVID-19-positive patients seemed to be more susceptible to tension and anxiety (65.74% vs. 61.43%), sleep problems (65.74% vs. 55.71%), and low spirits (65.10% vs. 48.57%) than the COVID-19-negative patients. Poor health was the top reason for mental health problems.

A cross-sectional investigation of 119 PD patients and 169 healthy controls in China has assessed the impact of COVID-19 on sleep and mental health by using the Pittsburgh Sleep Quality Index (PSQI) and the Hospital Anxiety and Depression Scale (HADS) [21]. During zero-COVID (year 2022), sleep disturbance was identified in 68.9% of PD patients, and PD patients had significantly higher scores of PSQI and HADS than healthy controls [21]. Another questionnaire study about the emotional and physiological symptoms of PD patients during the COVID-19 pandemic (year 2022) enrolled 94 PD patients and 188 controls [22]. It found that a significant number of PD patients were depressed and the major reason was COVID-19. Among the physiological symptoms, low energy, restlessness, and avoiding social interaction were significantly higher in PD patients [22]. Our data were consistent with previous studies. To our surprise, despite the high rate of emotional problems, the demands for professional psychological counseling and support were relatively low (20.29% during zero-COVID, 24.39% after zero-COVID). This may be explained by the low public awareness of mental illness and the immature status and low popularity of professional counseling in China [23]. More attention should be paid to the mental health problems in PD patients.

Our study also showed that PD patients tended to change their daily activities from offline to online, and spent less time on physical exercise during the pandemic. Moreover, the economic burden and caregiver’s burden had increased significantly. This might be related to the negative impacts of the shutdowns, which limited patients' outdoor activities, medical treatment seeking, and job seeking [24].

### Effect of COVID-19 on disease management of PD patients

In our study, 68.78% of PD patients reported reduced frequency of allopatric medical treatment due to the lockdown measures during zero-COVID. Patients tended to change their medical treatment from offline to online and most of them considered that their problems could be solved online (88.28% during zero-COVID, 87.12% after zero-COVID). Many PD patients would like to choose online rehabilitation guidance (69.56% during zero-COVID, 69.27% after zero-COVID) and purchase medications online (47.00% during zero-COVID, 26.63% after zero-COVID).

The COVID-19 pandemic has profoundly affected the delivery of medical care for PD patients, and digital rehabilitation strategies should be encouraged [25]. Although not superior to in-person visits, a growing body of evidence suggests that telemedicine services offer greater efficiency for PD patients [26]. An online survey in Italy has investigated the impact of COVID-19 on the access to telehealth care among 197 PD patients and 42 neurologists [27]. The results showed that 37.6% of PD patients and 88.1% of neurologists had chosen alternatives to in-person visits, while 13.7% of PD patients and 40.5% of neurologists used telemedicine services. Most of them were satisfied with the use of telemedicine during the COVID-19 pandemic, indicating that telemedicine has the potential to improve the care of PD patients, especially when access to in-person visits is limited [27]. In this COVID-19 era, sudden changes usually require a flexible adaptation to new circumstances. Telemedicine with digital visits, e-rehabilitation, and remote programming will break the obstacles of limited access to the routine visits to the hospital.

This survey-based study aimed to evaluate the impacts of COVID-19 on PD patients during and after zero-COVID in China. The study was mainly initiated in Shanghai and completed with the participation of multi-centers across the country, which would provide a comprehensive understanding of the effects of COVID-19 on PD patients from the Chinese database.

There are still several limitations of this study. First, although our study compared the situation during versus after zero-COVID, it was a cross-sectional study and did not follow up the same group of patients, so it lacked longitudinal data. Second, although online surveys are advantageous in providing large-scale and multicenter data, their reliability is hard to compare with offline investigation. The online survey was likely to bias towards milder COVID-19 cases as the PD patients who were able to complete a questionnaire might have recovered from the infection. There was a possibility of underestimating the proportion of death and severe disease. Third, the distribution of patients may be affected by the uneven development of regional networks and the education level of patients. It is likely that the availability of the online survey was limited to those with better technological skills or access to the necessary technology. Last, our survey only observed the influence of the COVID-19 pandemic on PD patients without mechanism exploration, which needs further studies in the future.

## Conclusions

In conclusion, COVID-19 aggravated the motor and non-motor symptoms of PD patients both during and after zero-COVID. Mental health problems, changes in daily activities, and increases in economic and caregiver burdens were also prominent. COVID-19 has changed the way of PD disease management, and demands for online medical treatment arose during the pandemic, including online rehabilitation and medication purchasing. Flexible adaptations of medical care delivery to patients under new circumstances are indispensable.

### Supplementary Information

**Additional file 1**. **Methods.** Details of the questionnaire design. **Table S1.** The demographics of PD participants during zero-COVID. **Table S2.** The demographics of PD participants after zero-COVID. **Fig. S1.** Symptoms reported by COVID-19 positive PD patients. **Fig. S2.** The mental health and demand for psychological support among PD patients during and after zero-COVID. **Fig. S3.** Changes of the time spent on physical exercise among PD patients during and after zero-COVID. **Fig. S4.** Changes of PD patients’ daily activity types during and after zero-COVID. **Fig. S5.** Changes of economic and caregiver’s burden during and after zero-COVID. **Fig. S6.** Changes of allopatric medical treatment frequency during and after zero-COVID. **Fig. S7.** Changes of offline medical treatment frequency during and after zero-COVID. **Fig. S8.** Changes of online medical treatment frequency during and after zero-COVID. **Fig. S9.** The effects of online medical treatment during and after zero-COVID. **Fig. S10.** Demand for remote rehabilitation during and after zero-COVID. **Fig. S11.** The effects of online rehabilitation during and after zero-COVID. **Fig. S12.** The willingness of continuation of online rehabilitation. **Fig. S13.** Demand for online drug purchase during and after zero-COVID.

## Data Availability

Anonymized data collected for the study and a data dictionary will be made available to other researchers following approval of a study proposal by SDC (chensd@rjh.com.cn) for 5 years from publication. The study protocol and statistical analysis plan are also available from SDC.

## Abbreviations

PD: Parkinson’s disease
COVID-19: Coronavirus disease 2019
SARS-CoV-2: Severe acute respiratory syndrome coronavirus 2
PSQI: Pittsburgh Sleep Quality Index
HADS: Hospital Anxiety and Depression Scale
