# Supplementary material for: Life and disease status of patients with Parkinson’s disease during and after zero-COVID in China: an online survey
Source: Transl Neurodegener. 2024 Feb 6;13:8. doi: 10.1186/s40035-024-00399-9 (PMC10845503; doi:10.1186/s40035-024-00399-9)
Supplement: Supplementary file 1 — Additional file 1. Methods. Details of the questionnaire design. Table S1. The demographics of PD participants during zero-COVID. Table S2. The demographics of PD participants after zero-COVID. Fig. S1. Symptoms reported by COVID-19 positive PD patients. Fig. S2. The mental health and demand for psychological support among PD patients during and after zero-COVID. Fig. S3. Changes of the time spent on physical exercise among PD patients during and after zero-COVID. Fig. S4. Changes of PD patients’ daily activity types during and after zero-COVID. Fig. S5. Changes of economic and caregiver’s burden during and after zero-COVID. Fig. S6. Changes of allopatric medical treatment frequency during and after zero-COVID. Fig. S7. Changes of offline medical treatment frequency during and after zero-COVID. Fig. S8. Changes of online medical treatment frequency during and after zero-COVID. Fig. S9. The effects of online medical treatment during and after zero-COVID. Fig. S10. Demand for remote rehabilitation during and after zero-COVID. Fig. S11. The effects of online rehabilitation during and after zero-COVID. Fig. S12. The willingness of continuation of online rehabilitation. Fig. S13. Demand for online drug purchase during and after zero-COVID. [file 40035_2024_399_MOESM1_ESM.docx]

**Additional File 1**

**Method S1** Details of the questionnaire design

**Table S1** The demographics of PD participants during zero-COVID

**Table S2** The demographics of PD participants after zero-COVID

**Figure S1** Symptoms reported by COVID-19 positive PD patients

**Figure S2** The mental health and demand for psychological support among PD patients during and after zero-COVID

**Figure S3** Changes of the time spent on physical exercise among PD patients during and after zero-COVID

**Figure S4** Changes of PD patients’ daily activity types during and after zero-COVID

**Figure S5** Changes of economic and caregiver’s burden during and after zero-COVID

**Figure S6** Changes of allopatric medical treatment frequency during and after zero-COVID

**Figure S7** Changes of offline medical treatment frequency during and after zero-COVID

**Figure S8** Changes of online medical treatment frequency during and after zero-COVID

**Figure S9** The effects of online medical treatment during and after zero-COVID

**Figure S10** Demand for remote rehabilitation during and after zero-COVID

**Figure S11** The effects of online rehabilitation during and after zero-COVID

**Figure S12** The willingness of continuation of online rehabilitation

**Figure S13** Demand for online drug purchase during and after zero-COVID

**Method S1 Details of the questionnaire design**

To understand how the COVID-19 pandemic affected PD patients, the survey was mainly divided into four sections, corresponding to our aims: (1) general status of COVID-19 infection among PD patients; (2) impact of the COVID-19 pandemic on the PD motor and non-motor symptoms; (3) impact of the COVID-19 pandemic on the PD daily life and social function changes; and (4) impact of the COVID-19 pandemic on the PD disease managements.

For our first aim, we evaluated COVID-19 infection related symptoms, classification and proportion of disease severity, hospitalization and death rate. COVID-19 infection severity classification (mild, medium, serious, extremely serious) refers to “novel coronavirus infection diagnosis and treatment plan (trial 10th edition)”.

For our second aim, we assessed the status of PD patients’ motor and non-motor symptoms during and after zero-COVID. Motor symptoms mainly focused on falls, turning over difficulty, balance instability, bradykinesia, tremor and rigidity. Non-motor symptoms mainly included emotion, cognition, sleep, somatosensory, autonomic nervous function and others. The proportion of aggravated motor and non-motor symptoms was investigated.

For our third aim, we evaluated changes of mental psychology, daily activity, economic and caregiver burdens of PD patients during and after zero-COVID. In the mental psychology part, PD patients’ attitudes toward COVID-19 pandemic and mental states during the pandemic were surveyed, and reasons for mental and psychological problems were also analyzed. In the daily activity part, changes of time spent on physical exercise and changes of daily online and offline activities (including indoor activities, community activities, short/long distance trip, offline/online disease education, offline/online patients’ communication) were concerned. In the economic and caregiver burden part, changes of patient and family income and expenditure, and changes of the caregivers’ burdens were investigated.

For our fourth aim, we evaluated changes of allopatric medical treatment, offline and online medical treatment, online rehabilitation and online drug purchase. The survey mainly focused on changes of the way of medical treatment, the frequency of different forms of medical treatment, the experience, satisfaction, and the expectation of the new way of medical treatment.

**Table S1** **The demographics of PD participants during zero-COVID**

|  | Whole patients  (n=1764) | Lockdowned patients  (n=200) | Unlockdowned patients  (n=1564) | *P* | |
| --- | --- | --- | --- | --- | --- |
| Age, mean (SD), y | 62.52 (10.47) | 60.81 (10.50) | 62.74 (10.44) | | 0.066 |
| Gender, No. (M/F) | 899/865 | 107/93 | 792/772 | | 0.446 |
| Disease duration, mean (SD), y | 7.00 (5.00) | 7.00 (5.00) | 7.00 (5.00) | | 1.000 |
| Vaccination status (%) |  |  |  | | 0.140 |
| 0 | 26.70% | 28.50% | 26.47% | |  |
| 1 | 4.20% | 3.00% | 4.35% | |  |
| 2 | 19.90% | 25.00% | 19.25% | |  |
| ≥3 | 49.21% | 43.50% | 49.94% | |  |

PD, Parkinson’s disease; COVID-19, the coronavirus disease 2019; *P* indicates comparison between lockdowned and unlockdwoned patients.

**Table S2 The demographics of PD participants after zero-COVID**

|  | Whole patients  (n=537) | COVID-19 positive patients  (n=467) | COVID-19 negative patients  (n=70) | | *P* |
| --- | --- | --- | --- | --- | --- |
| Age, mean (SD), y | 63.48 (9.93) | 63.45 (10.04) | | 63.71 (9.24) | 0.838 |
| Gender, No. (M/F) | 276/261 | 242/225 | | 34/36 | 0.612 |
| Disease duration, mean (SD), y | 8.85 (5.64) | 8.83 (5.65) | | 8.93 (5.61) | 0.890 |
| Vaccination status (%) |  |  | |  | 0.268 |
| 0 | 29.98% | 29.76% | | 31.43% |  |
| 1 | 12.10% | 12.85% | | 7.14% |  |
| 2 | 13.97% | 13.06% | | 20.00% |  |
| ≥3 | 43.95% | 44.33% | | 41.43% |  |

PD, Parkinson’s disease; COVID-19, the coronavirus disease 2019; *P* indicates comparison between COVID-positive and COVID-19 negative patients.


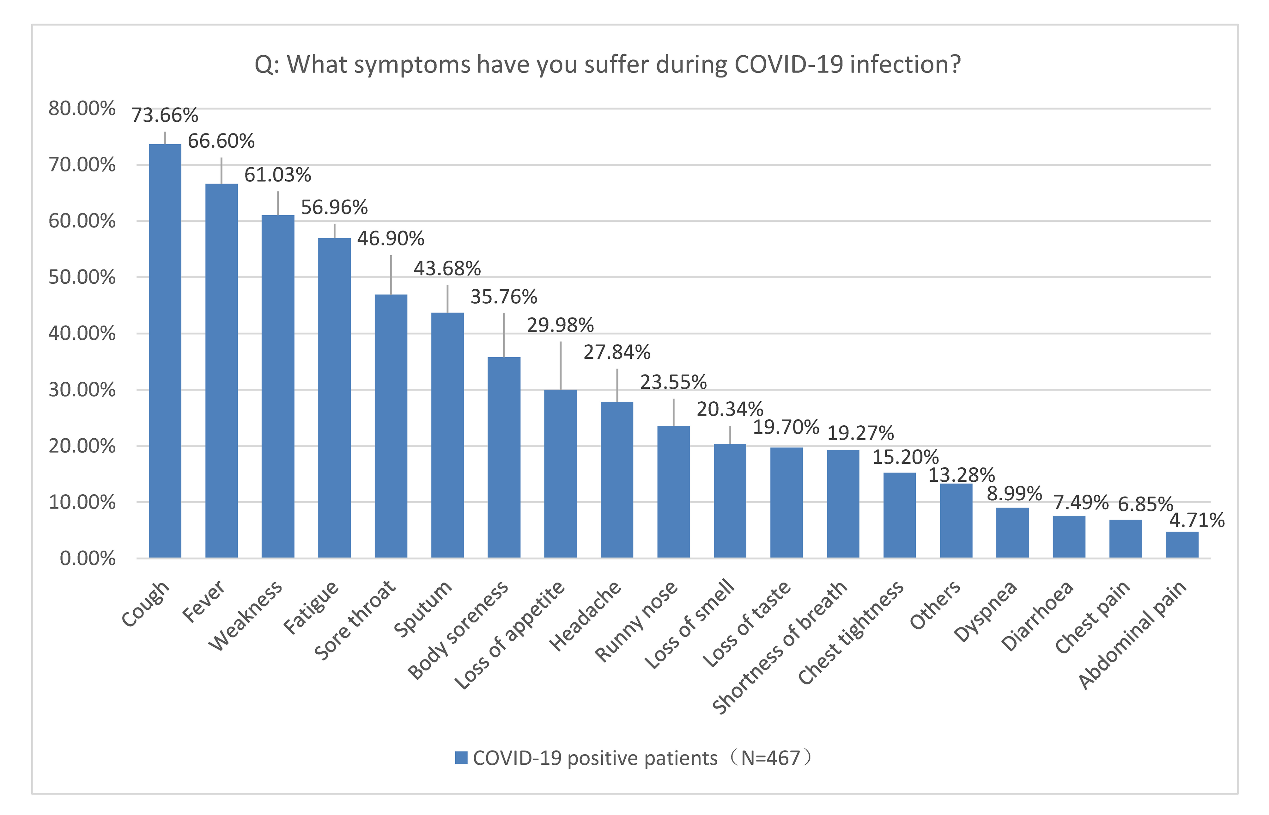


**Figure S1 Symptoms reported by COVID-19 positive PD patients**


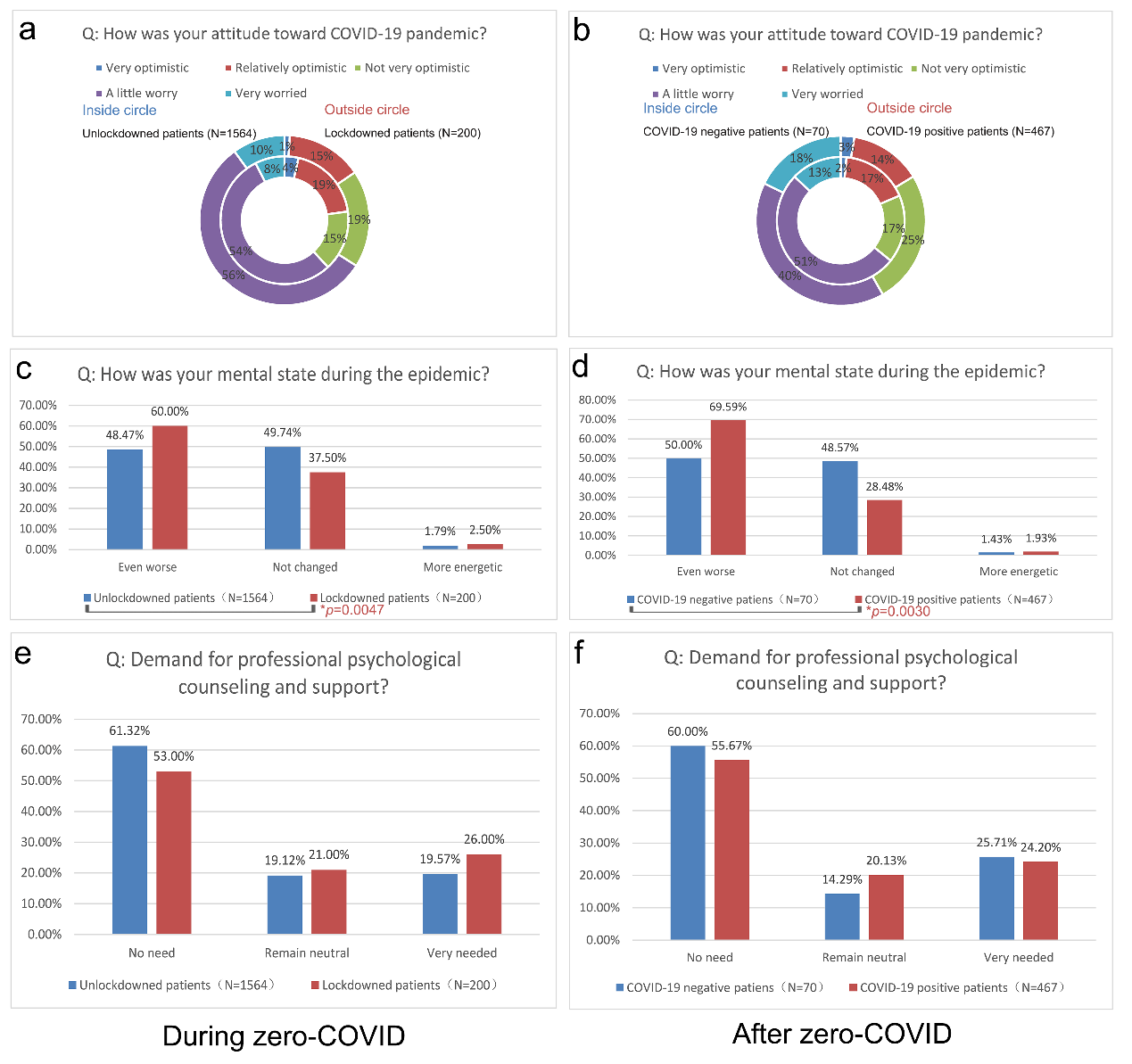


**Figure S2 The mental health and demand for psychological support among PD patients during and after zero-COVID. a** PD patients’ attitudes towards COVID-19 pandemic during zero-COVID. Inside circle represents patients with lockdown experiences and outside circle represents patients without lockdown experiences. During zero-COVID, 62.36% patients felt worried about the pandemic while only 21.99% patients felt optimistic. The patients with lockdown experiences showed relatively higher rate of worry than the patients with no lockdown experience (66.00% vs. 61.89%, χ^2^=8.37, p=0.079). **b** PD patients’ attitudes towards COVID-19 pandemic after zero-COVID. Inside circle represents COVID-19 negative patients and outside circle represents COVID-19 positive patients. COVID-19-negative patients showed relatively higher rate of worry than COVID-19-positive patients (64.29% vs. 58.24%, χ^2^=5.17, p=0.270), probably because that the negative patients were more worried to be infected. **c** 49.77% of the patients reported worsening of mental state, and the situation was more serious among the patients with lockdown experiences than those without (60.00% vs. 48.47%, χ^2^=10.7, p=0.0047). **d** 67.04% of the patients had worsening of mental problems than the past, and the situation was more serious among COVID-19-positive patients than negative patients (69.59% vs. 50.00%, χ^2^=11.5, p=0.003). **e** 20.29% of the PD patients demanded for professional psychological counseling and support, and the patients with lockdown experiences had especially higher demands than the patients without lockdown experiences (26.00% vs. 19.57%, χ^2^=5.97, p=0.0504). **f** 24.39% of the PD patients demanded for professional psychological counseling and support, and COVID-19-positive patients had similar demands as COVID-19-negative persons (24.20% vs. 25.71%, χ^2^=1.33, p=0.514).


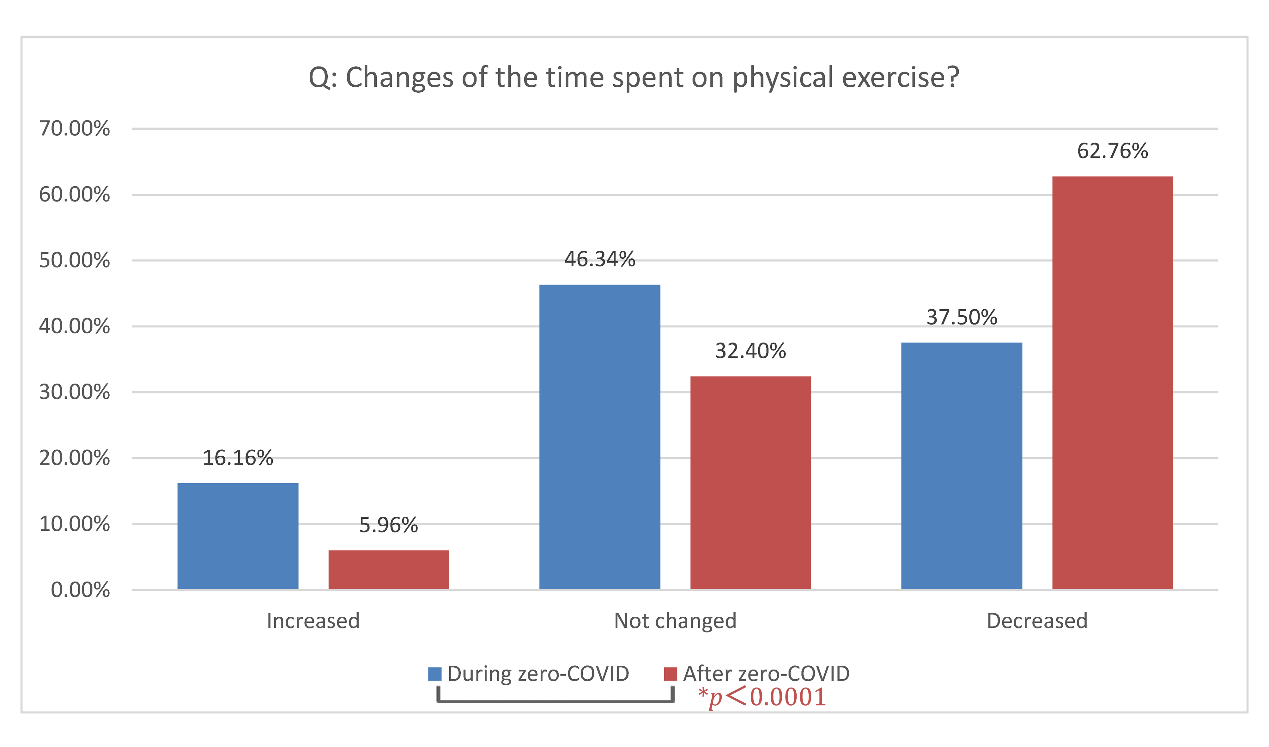


**Figure S3 Changes of the time spent on physical exercise among PD patients during and after zero-COVID.**


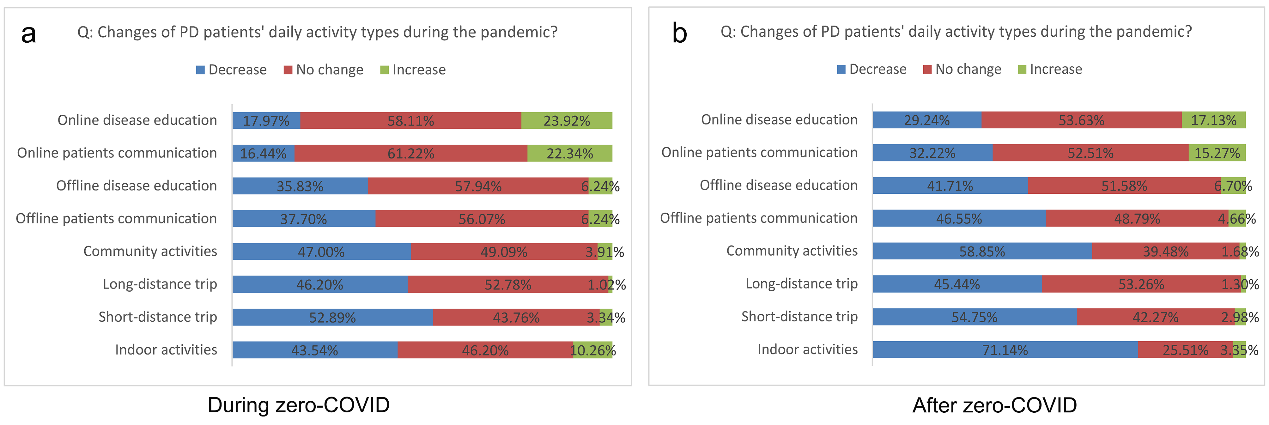


**Figure S4 Changes of PD patients’ daily activity types during and after zero-COVID. a** Changes of PD patients’ daily activity types during zero-COVID. **b** Changes of PD patients’ daily activity types during zero-COVID.


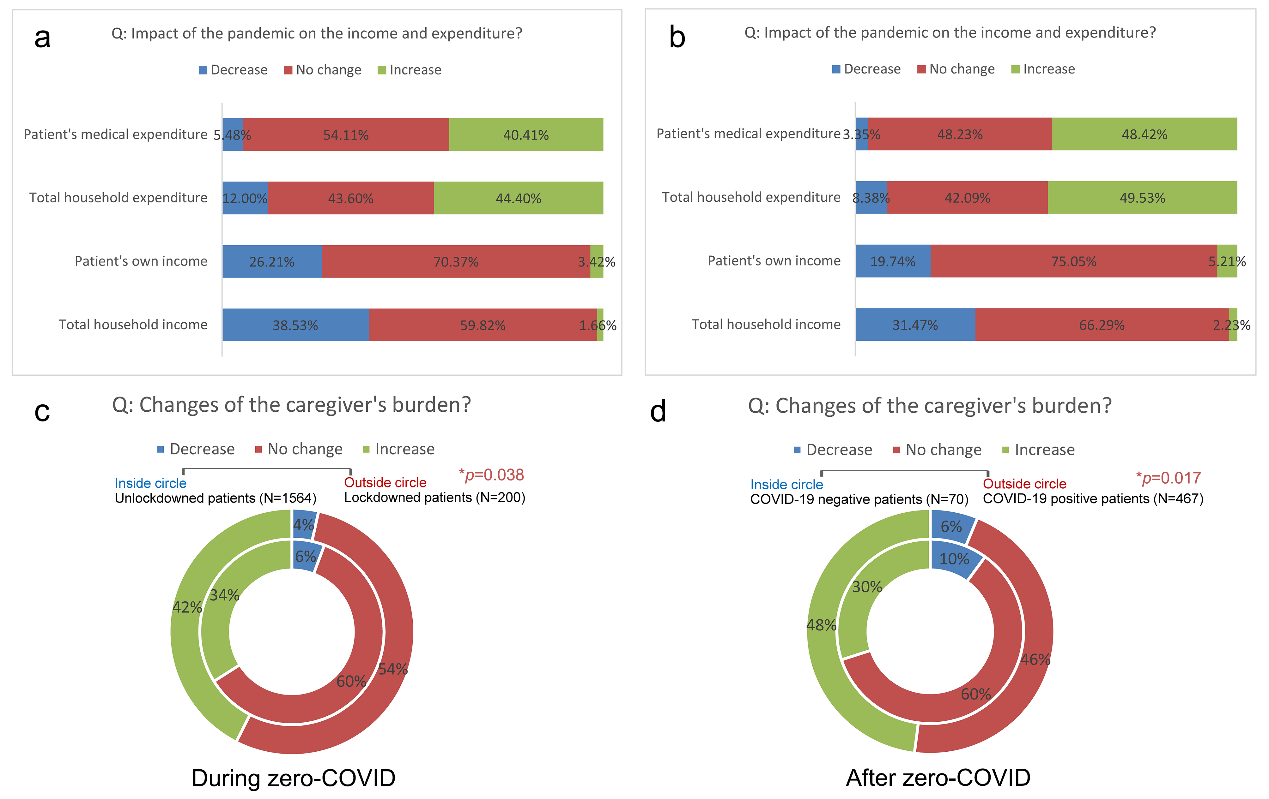


**Figure S5 Changes of economic and caregiver’s burden during and after zero-COVID. a** Impact of COVID-19 pandemic on the income and expenditure of PD patients and their families during zero-COVID. **b** Impact of COVID-19 pandemic on the income and expenditure of PD patients and their families after zero-COVID. **c** Changes of caregiver’s burden during zero-COVID. Inside circle represents patients with lockdown experiences and outside circle represents patients without lockdown experiences. During zero-COVID, 34.92% of the caregivers complained about the increase of caring burden, and the burdens were much heavier among those caregivers of lockdowned PD patients compared with those of unlockdowned populations (42.50% vs. 33.95%, χ^2^=6.53, p=0.038). **d** Changes of caregiver’s burden after zero-COVID. Inside circle represents COVID-19-negative patients and outside circle represents COVID-19-positive patients. After zero-COVID, 45.62% of the caregivers complained about the increase of caring burden, and the burdens were much heavier among those caregivers of COVID-19 positive PD patients compared with those of COVID-19 negative populations (47.97% vs. 30.00%, χ^2^=8.18, p=0.017).


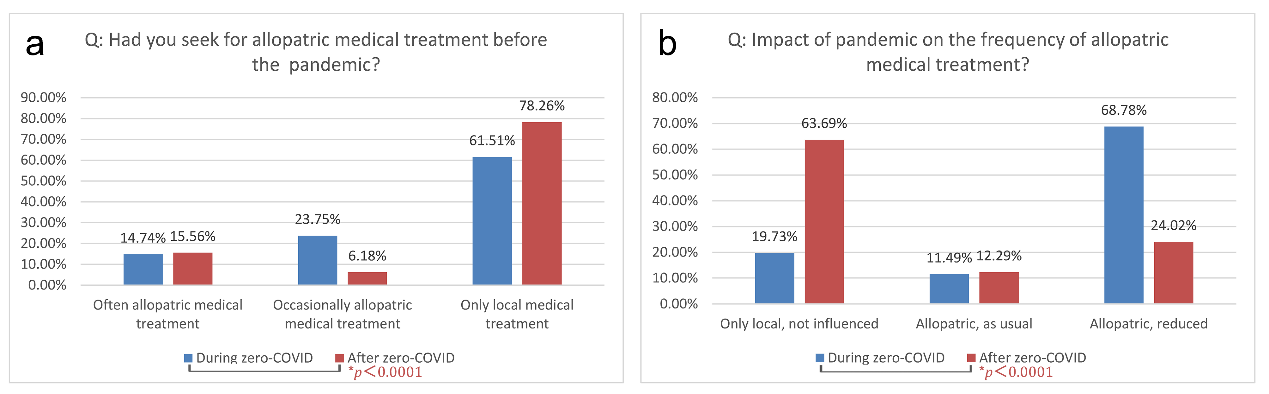


**Figure S6 Changes of allopatric medical treatment frequency during and after zero-COVID. a** The frequency of allopatric medical treatment for PD patients before COVID-19 pandemic (during zero-COVID vs. after zero-COVID, χ^2^=266.9, p <0.0001). **b** Change of the frequency of allopatric medical treatment for PD patients during COVID-19 pandemic (during zero-COVID vs. after zero-COVID, χ^2^=893.2, p <0.0001).


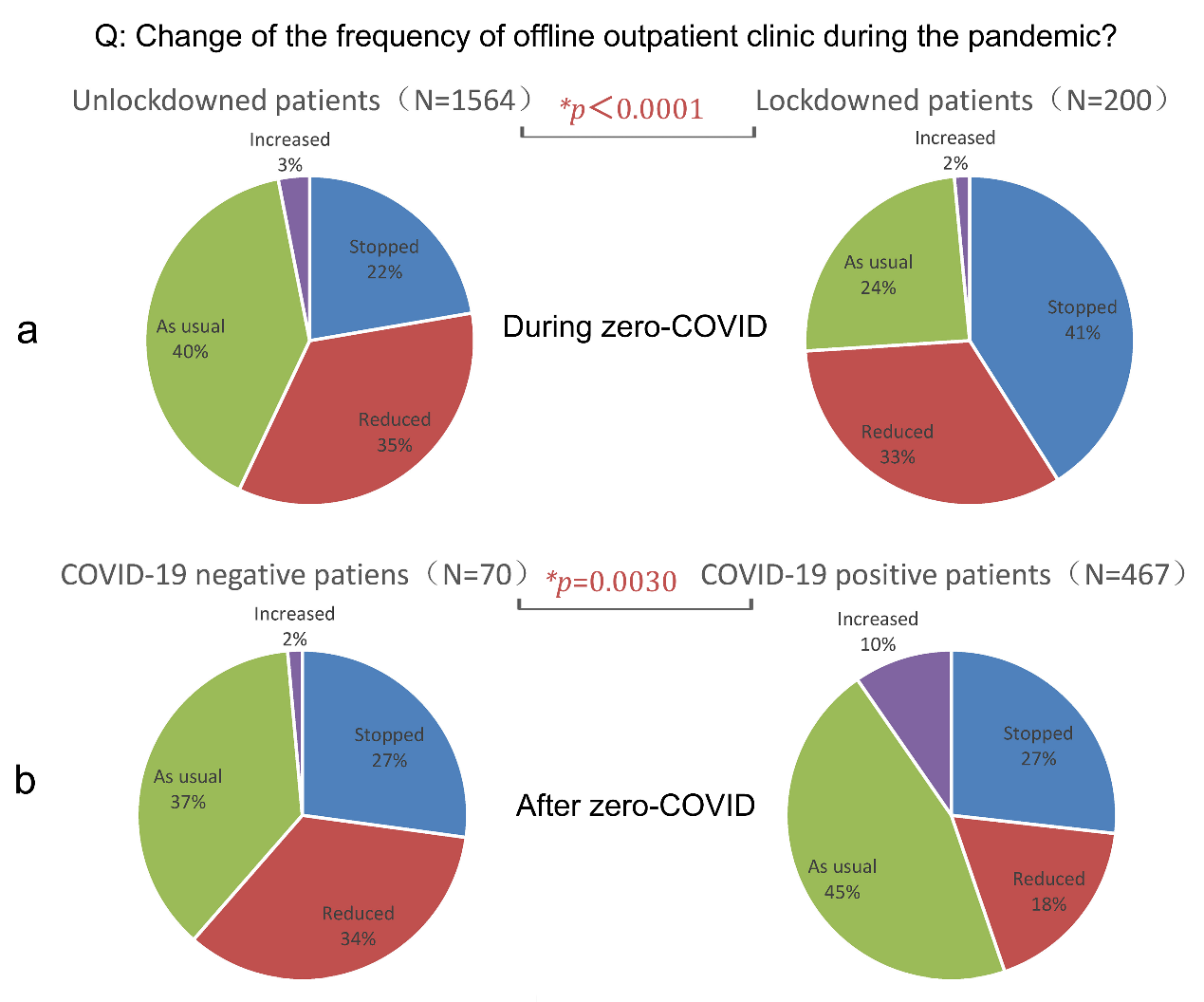


**Figure S7 Changes of offline medical treatment frequency during and after zero-COVID. a** Change of the frequency of offline outpatient clinic during zero-COVID. During zero-COVID, 24.60% of PD patients stopped visiting outpatient clinic offline and 34.52% of patients had reduced frequency of offline outpatient clinic. This situation was even worse in the lockdowned patients than the unlockdowned ones (stopped: 41.00% vs. 22.31%, χ^2^=38.0, p＜0.0001). **b** Change of the frequency of offline outpatient clinic after zero-COVID. After zero-COVID, 26.82% of PD patients stopped visiting outpatient clinic offline and 20.11% of patients had reduced frequency of offline outpatient clinic. However, 44.51% of patients visited the offline outpatient clinic as usual. COVID-19 negative patients showed remarkable decrease in offline outpatient clinic visiting than COVID-19 positive patients (reduced: 34.29% vs. 17.99%, χ^2^=13.8, p=0.003).


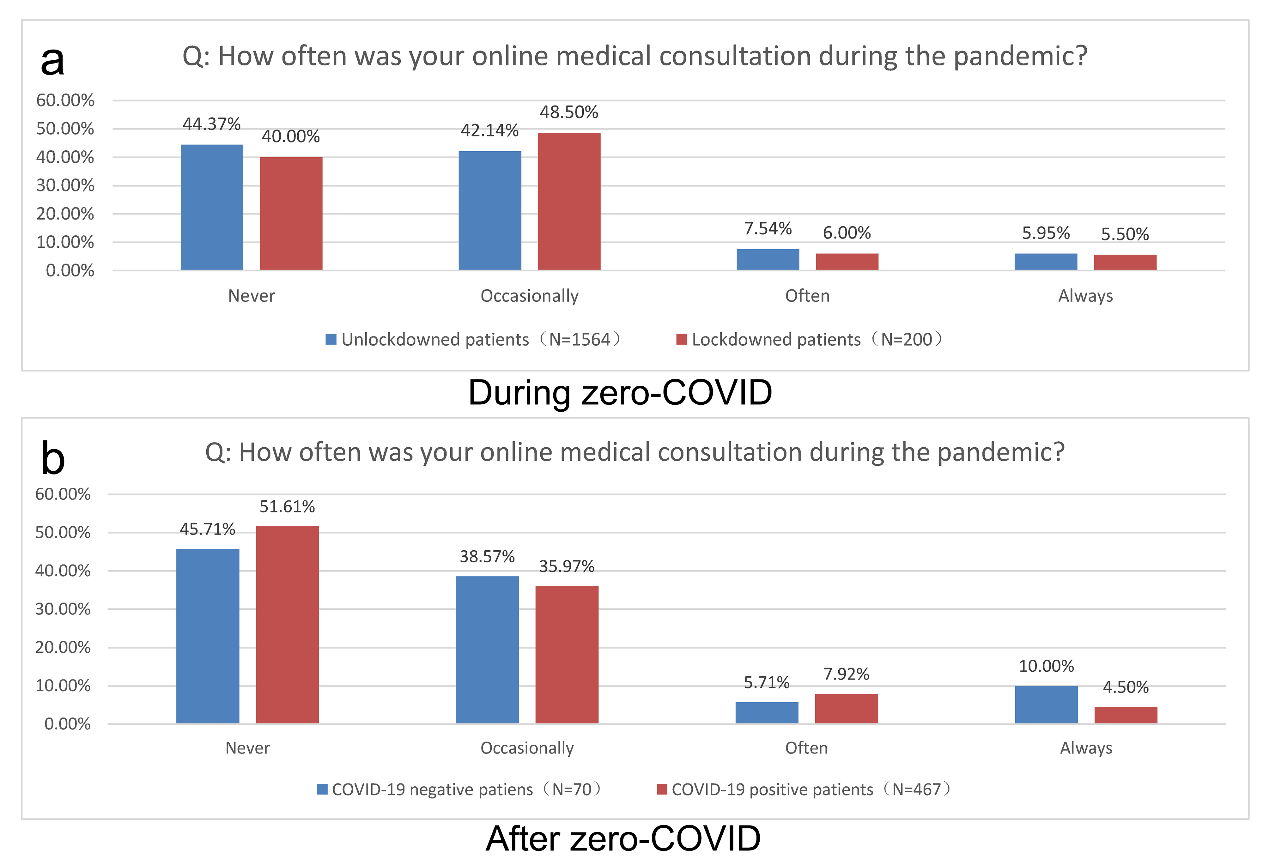


**Figure S8** **Changes of online medical treatment frequency during and after zero-COVID. a** The frequency of online medical treatment during zero-COVID. 56.24% of PD patients had chosen online medical consultation during zero-COVID. The ratio of online medical consultation experience was relatively higher in the lockdowned patients than the unlockdowned ones (60.00% vs. 55.63%, χ^2^=3.08, p=0.379). **b** The frequency of online medical treatment after zero-COVID. After zero-COVID, 49.16% of PD patients had chosen online medical consultation. The ratio of online medical consultation experience was relatively higher in the COVID-19 negative patients than the COVID-19 positive ones (54.29% vs. 48.39%, χ^2^=4.45, p=0.217).


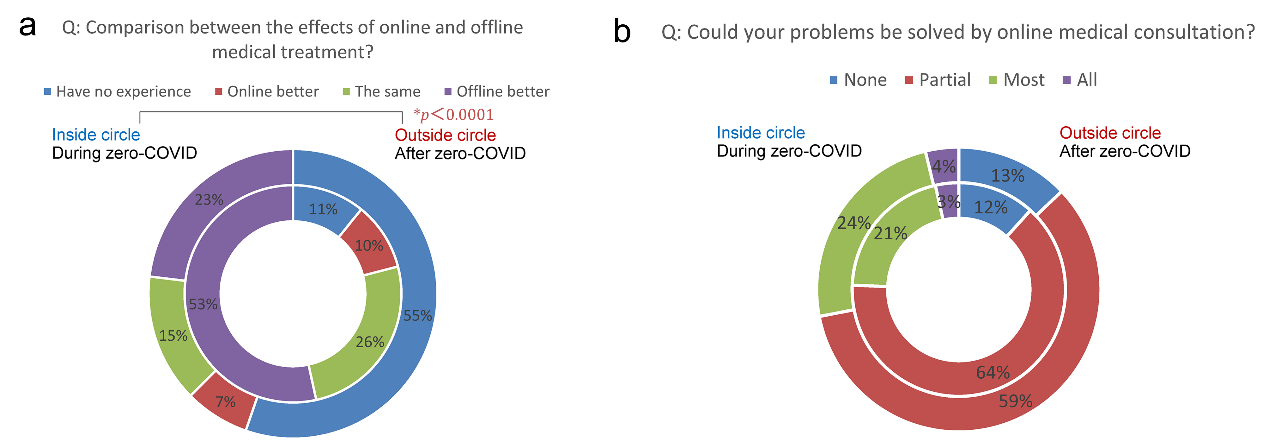


**Figure S9 The effects of online medical treatment during and after zero-COVID. a** Comparison between the effects of online and offline medical treatment. During zero-COVID, 10.12% of patients thought online medical treatment was better than the offline outpatient clinic, 25.66% of patients received the same effects, 53.44% of patients insisted on better effects of offline outpatient clinics 11% of patients had no experience of online consultation. After zero-COVID, 7.26% of patients thought online medical consultation was better than the offline outpatient clinic, 14.34% of patients received the same effects, 23.09% of patients insisted on better effects of offline outpatient clinics, 55% of patients had no experience of online consultation (during zero-COVID vs. after zero-COVID, χ^2^=883.7, p＜0.0001). **b** Investigation on the ability of online medical treatment to solve PD patients' problems during COVID-19 pandemic. For those who had experience of online medical consultation during the pandemic, most (88.28% during zero-COVID, 87.12% after zero-COVID) of patients considered that their problems could be solved. Inside circle represents during zero-COVID and outside circle represents after zero-COVID both in figure a and b.


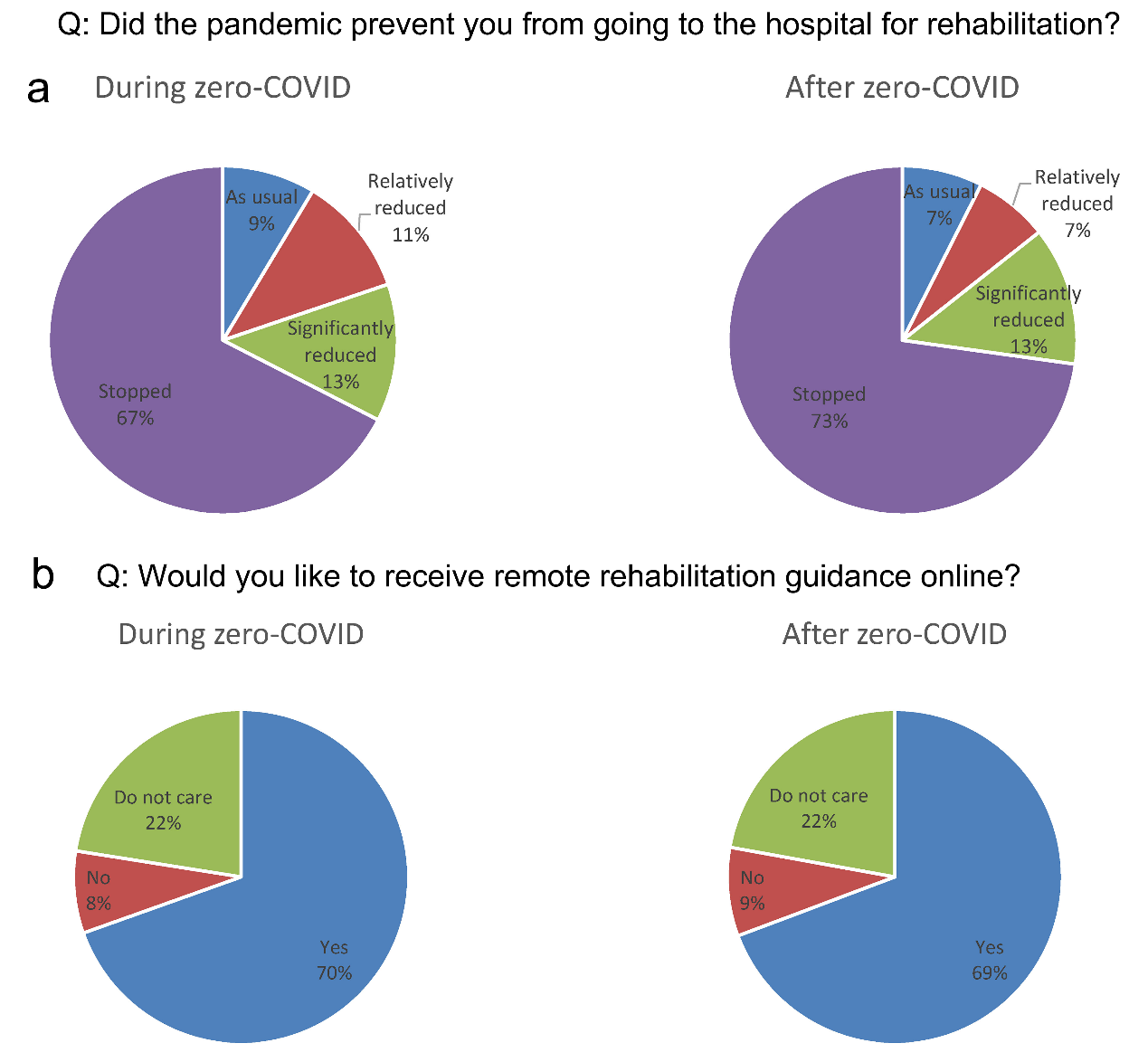


**Figure S10 Demand for remote rehabilitation during and after zero-COVID. a** The influence of COVID-19 pandemic on preventing PD patients from going to hospital for rehabilitation. During zero-COVID, the pandemic stopped 67.46% of PD patients from rehabilitation, 12.76% of patients had significantly reduced rehabilitation, and 11.17% of patients received relatively less rehabilitation in hospitals. After zero-COVID, the pandemic stopped 72.81% of PD patients from rehabilitation, 12.85% of patients had significantly reduced rehabilitation, and 6.89% of patients received relatively less rehabilitation in hospitals. **b** PD patients’ willingness to receive remote rehabilitation guidance online. 69.56% of PD patients during zero-COVID and 69.27% of PD patients after zero-COVID would like to choose remote rehabilitation guidance online.


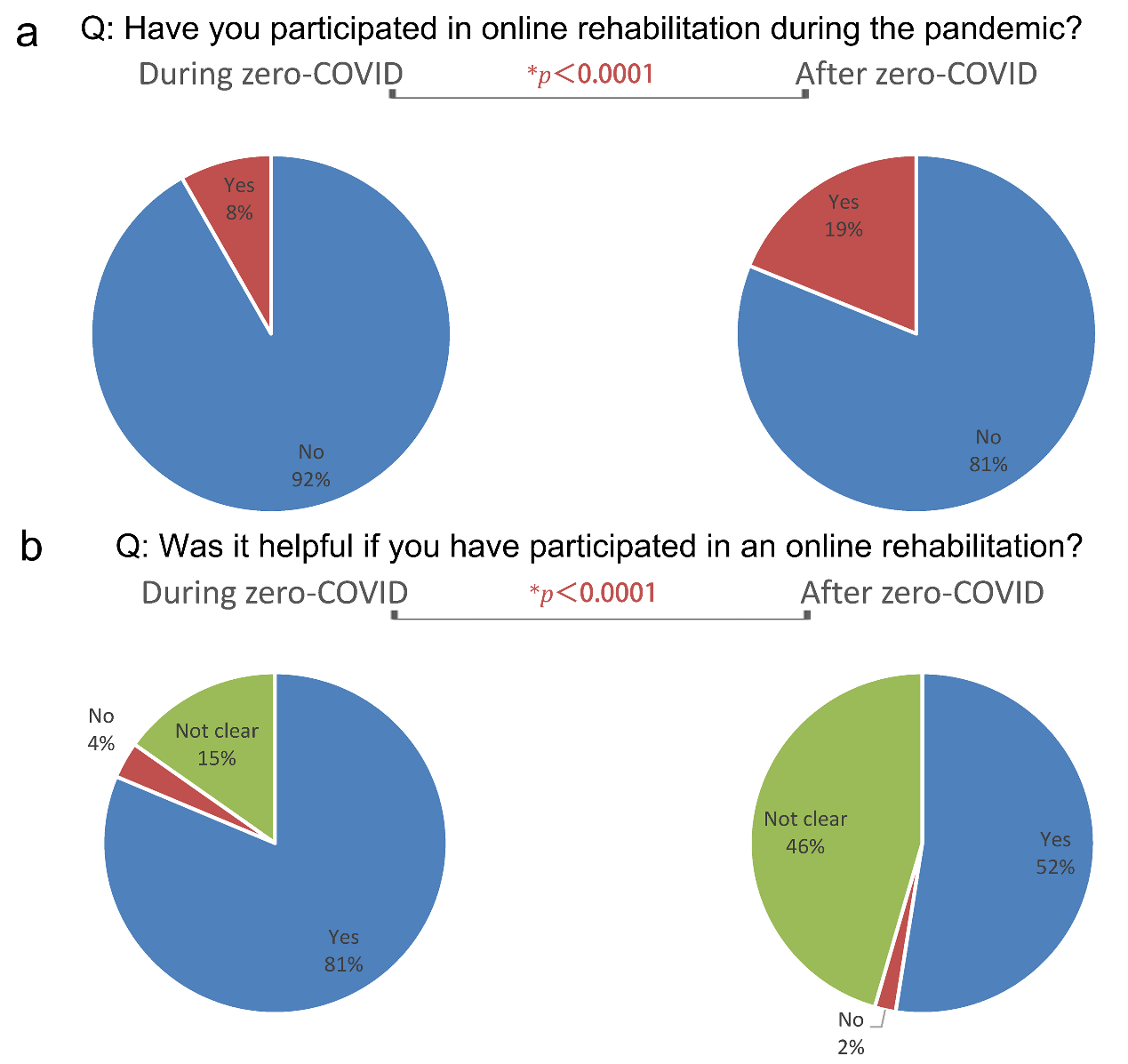


**Figure S11** **The effects of online rehabilitation during and after zero-COVID. a** Investigation on the experience of online rehabilitation in PD patients during COVID-19 pandemic. 8.22% of patients had already experienced the online rehabilitation guidance during zero-COVID. After zero-COVID, 18.81% of PD patients had already experienced the online rehabilitation guidance. **b** Investigation on the benefits of online rehabilitation for PD patients during COVID-19 pandemic. During zero-COVID, 81.38% of the participants found online rehabilitation helpful to themselves. After zero-COVID, 52.48% of the participants found online rehabilitation helpful to themselves.


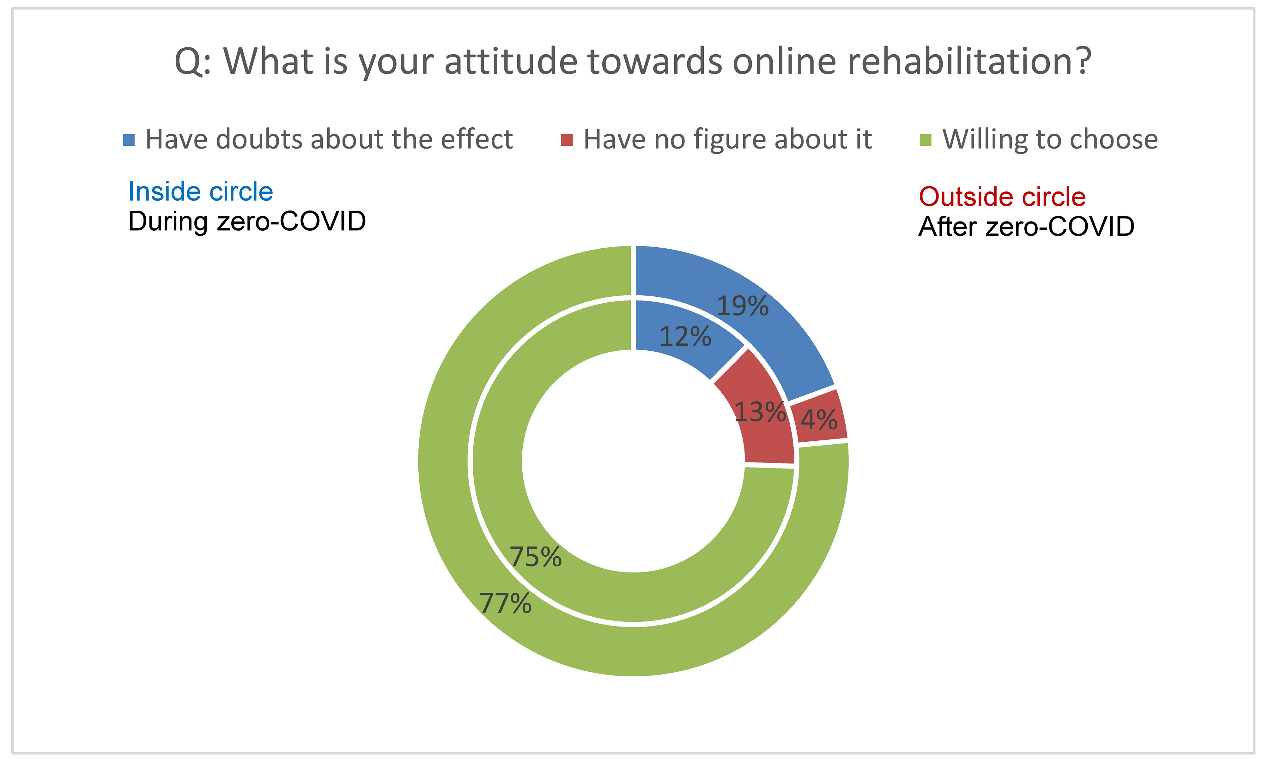


**Figure S12 The willingness of continuation of online rehabilitation.** Inside circle represents during zero-COVID and outside circle represents after zero-COVID. During zero-COVID, 74.48% of patients were willing to continue to choose online rehabilitation. After zero-COVID, 76.53% of patients were willing to continue to choose online rehabilitation.


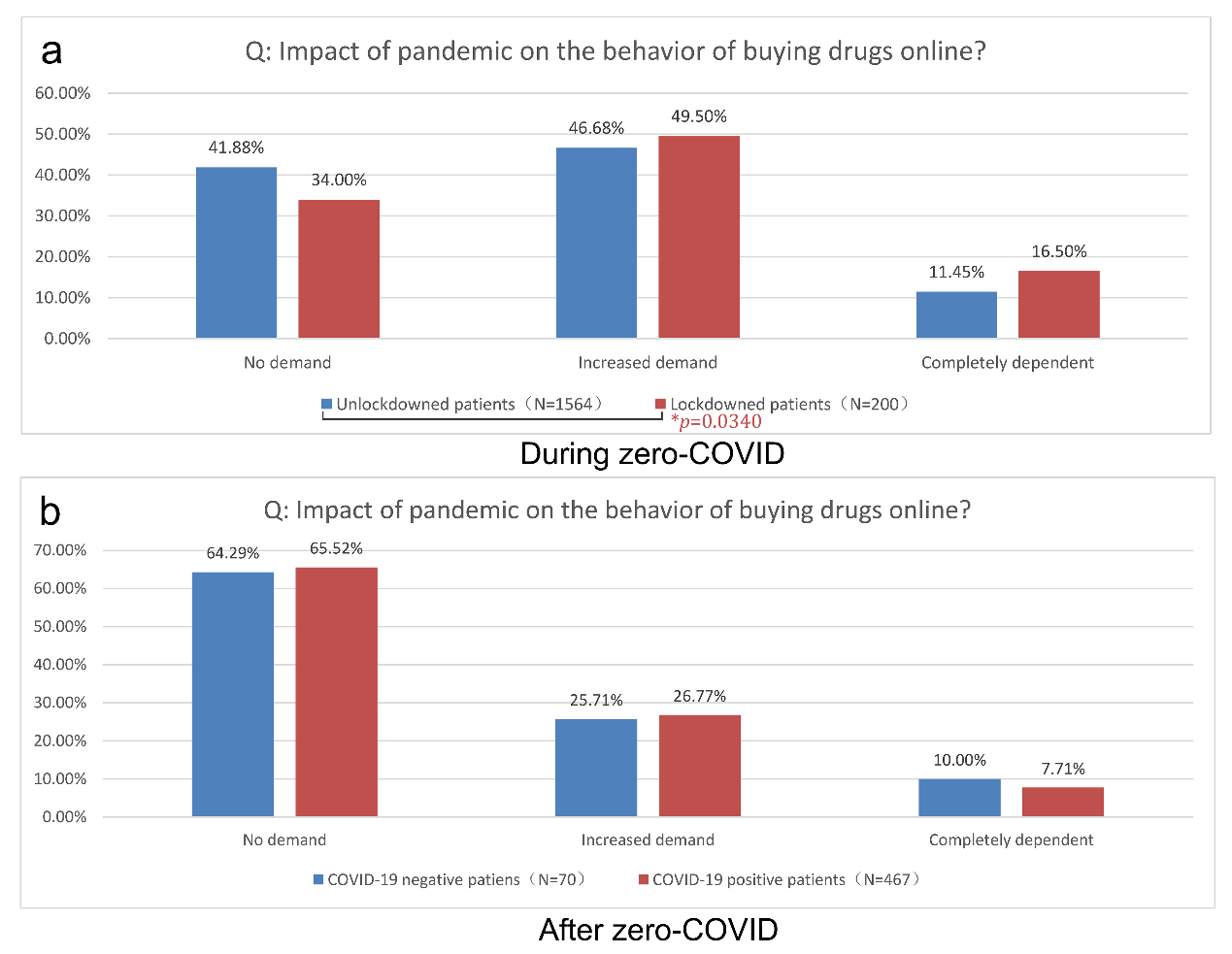


**Figure S13 Demand for online drug purchase during and after zero-COVID. a** Demands of PD patients for buying drugs online during zero-COVID. **b** Demands of PD patients for buying drugs online after zero-COVID.
